# The Outcome of Cholangitis After Percutaneous Biliary Drainage in Neoplastic Jaundice

**DOI:** 10.1155/1993/17078

**Published:** 1993

**Authors:** Riccardo A. Audisio, Carlo Morosi, Federico Bozzetti, Guido Cozzi, Massimo Bellomi, Paola Pisani, Alessandra Pestalozza, Leandro Gennari, Aldo Severini

**Affiliations:** 1 Surgical Oncology D Istituto Nazionale per lo Studio e la Cura dei Tumori via Venezian, 1 Milano 20133 Italy; 2 Gastrointestinal Radiology Section Istituto Nazionale per lo Studio e la Cura dei Tumori Milano Italy; 3 Surgical Oncology A Istituto Nazionale per lo Studio e la Cura dei Tumori Milano Italy; 4 Istituto di Scienze Radiologiche Universita' di Milano Italy; 5 Epidemiology Unit Istituto Nazionale per lo Studio e la Cura dei Tumori Milano Italy

## Abstract

The purpose of this paper is to evaluate factors affecting the outcome of cholangitis after PTBD in jaundiced cancer patients. Twenty nine patients with neoplastic jaundice (male/female ratio 13/16, median age 55 years) with full clinical data, were treated by PTBD and developed cholangitis at a median of 9 days later. Four patients (14%) died of biliary sepsis a median of one month after PTBD while the other 25 survived a median of 6 months, with one week median duration of cholangitis. The probability of the cholangitis resolving was analyzed by time to resolution and it was found that 50% and 100% of the recoveries occurred 5 and 9 months respectively from the onset of the complication.

The series was analyzed to determine the role of several variables (disease/patient/treatment related) in the resolution of cholangitis. Only a low stricture site, a large initial drainage catheter (10F) and a temperature increase exceeding 39^°^ C were correlated with a positive outcome. We conclude that PTBD-related cholangitis has, in our experience, a good chance of cure, low mortality rate and satisfactory 6 months median survival.


*HPB Surgery*, 1993, Vol. 6, pp. 287–293
Reprints available directly from the publisher
Photocopying permitted by license only

© 1993 Harwood Academic Publishers GmbH
Printed in the United States of America

### THE OUTCOME OF CHOLANGITIS AFTER PERCUTANEOUS BILIARY DRAINAGE IN NEOPLASTIC JAUNDICE

[1]RICCARDO A. AUDISIO, [2]CARLO MOROSI, [3]FEDERICO BOZZETTI, [2]GUIDO COZZI, [4]MASSIMO BELLOMI, [5]PAOLA PISANI, [2]ALESSANDRA PESTALOZZA, [3]LEANDRO GENNARI and [2]ALDO SEVERINI

*[1]Surgical Oncology D, [2]Gastrointestinal Radiology Section, [3]Surgical Oncology A, [5]Epidemiology Unit, Istituto Nazionale per lo Studio e la Cura dei Tumori, Milano and [4]Istituto di Scienze Radiologiche, Universita'di Milano*

*(Received 19 May 1992)*

The purpose of this paper is to evaluate factors affecting the outcome of cholangitis after PTBD in jaundiced cancer patients. Twenty nine patients with neoplastic jaundice (male/female ratio 13/16, median age 55 years) with full clinical data, were treated by PTBD and developed cholangitis at a median of 9 days later. Four patients (14%) died of biliary sepsis a median of one month after PTBD while the other 25 survived a median of 6 months, with one week median duration of cholangitis. The probability of the cholangitis resolving was analyzed by time to resolution and it was found that 50% and 100% of the recoveries occurred 5 and 9 months respectively from the onset of the complication.

The series was analyzed to determine the role of several variables (disease/patient/treatment related) in the resolution of cholangitis. Only a low stricture site, a large initial drainage catheter (10F) and a temperature increase exceeding 39° C were correlated with a positive outcome. We conclude that PTBD-related cholangitis has, in our experience, a good chance of cure, low mortality rate and satisfactory 6 months median survival.

KEY WORDS: Biliary drainage, jaundice, PTBD, cholangitis

Although recent experience[1–3] suggests a decrease in septic problems after percutaneous transhepatic biliary drainage (PTBD), cholangitis still occurs with a median prevalence of 30%. Some factors were associated with a higher risk of cholangitis due to PTBD, in a previous report of ours[4], but little is known about factors influencing the clinical outcome in these patients. In an attempt to answer this question, we examined retrospectively the clinical data of 29 episodes of cholangitis after PTBD.

### PATIENTS AND METHODS

Our Institutional policy for dealing with obstructive jaundice is: Percutaneous Transhepatic Cholangiography (PTC) to provide accurate images, followed if

Address correspondence to: R.A. Audisio, Surgical Oncology D, Istituto Nazionale per lo Studio, e la Cura dei Tumori, via Venezian, 1, 20133 Milan, Italy.

necessary by Percutaneous Transhepatic Biliary Drainage (PTBD); this will reduce bilirubin preoperatively (5) with an improvement in hepatic regeneration and Kupffer cell response, and it provides definitive treatment if there is no surgical option. In the period from 1985 to 1989, 306 PTBDs were performed at the Istituto Nazionale Tumori of Milan.

We define cholangitis or recurrent cholangitis as a single or recurrent septic event with spiking fever exceeding 38.5° C, weakness sometimes accompanied by prostration, chills, hypotension and no sign of sepsis elsewhere.

The purpose of this paper is to define the mortality rate of cholangitis and the prognostic factors associated with a positive outcome. The incidence of cholangitis was 17.5% in cancer patients and 10.5% in non cancer patients with a total of 46 occurrences, six had inadequate clinical records seven had non neoplastic strictures and four had had major liver surgery so that their clinical septic course could be confused with postoperative subphrenic abscess, leaving only 29 occurrences in 28 patients for the present study.

In present series of 29 cancer patients (13 males and 16 females, median age 55 years, range 32 to 73 years), all had a histologically proven neoplastic stricture due to: primary liver cancer (10) or liver metastasis from colorectal cancer (9), gastric cancer (4), breast cancer (1) or gynecological malignancy (1); 4 subjects had pancreatic cancer and two of these had synchronous liver metastases. PTBD was the only procedure used to treat these patients who were poor candidates for other forms of palliation.

Data defining the patients' general condition, biochemistry, aetiology of jaundice and characteristics of the stricture are given in Table 1. Table 2 gives information about PTBD itself as well as procedures undertaken to improve bile flow through the catheter. Eighteen patients had an 8F and 11 had a 10F catheter. At the onset of cholangitis, it was externally opened in 7 patients and closed in 22, its tip passing through and below the stricture.

**Table 1** Characteristics of the series before the occurrence of cholangitis

| *Variables* | *N. patients* | *Median* | *(Range)* |
|---|---|---|---|
| Demographic/clinical | | | |
| sex M/F | 13/16 | | |
| age (years) | | 55 | (32–73) |
| perform status* 1 | 13 | | |
| 2 | 15 | | |
| 3 | 1 | | |
| Tumor related | | | |
| Primary liver carcinoma | 10 | | |
| liver metastasis | 17 | | |
| pancreatic cancer | 2 | | |
| above/at confluence | 22 | | |
| below " | 7 | | |
| duodenal invasion or infiltration | 4 | | |
| Biochemical | | | |
| basal WB count/mL | | 7400 | (3200–16000) |
| Total bilirubin mgs% | | 14 | (3–34) |

* according to the ZUBROD-WHO scale

**Table 2** Variables recorded at the occurrence of cholangitis and after it

| *Variables* | | *N. patients* |
|---|---|---|
| At the onset of cholangitis | | |
| onset from PTBD≤8 days | | 13 |
| >8 days | | 16 |
| drainage external | | 7 |
| internal | | 22 |
| drainage diameter | 8F | 18 |
| | 10F | 11 |
| | | 8 |
| drainage obstruction* no | | 21 |
| temperature ≤39° | | 22 |
| >39° | | 7 |
| WB count/ml ≤18.100 | | 15 |
| >18.100 | | 14 |
| After the onset of cholangitis | | |
| number of | 1 | 15 |
| exchanges | >1 | 14 |
| maximum diameter ≤12 F | | 24 |
| employed | >F | 5 |
| wash-outs employed | yes | 16 |
| | no | 13 |
| brushings | yes | 4 |
| | no | 25 |
| externally opened | yes | 25 |
| | no | 4 |
| broad spectrum antibiotics | | 19 |
| specific anbitioticsb | | 10 |
| systemic antibiotics | | 22 |
| systemic+intrabiliary | | 7 |
| injected antibiotics according to the lab resistence tests | | |
| n. days of antibiotic ≤13 | | 18 |
| therapy | >13 | 11 |

* reasological evidence.

At the onset of cholangitis median body temperature was 39 °C (range 38.5--40) and the median white cell count was 18100 /mL (7800–35000); bile was cultured and antibiotic sensitivities determined. Therapeutic antibiotics were given for a median of 13 days (range 5–40).

A third generation cephalosporin was administered to nineteen subjects; the other ten were given specific antibiotics according to sensitivity tests. Twenty-two patients were given intravenous antibiotics and seven had the drug injected into the biliary tree through the catheter. Drainage catheters were changed once in 15 patients and more than once in 14 (median 3, range 2–11).

Follow-up time was a median of four months (range 1–27 months) from PTBD until death.

The duration of cholangitis was defined as the number of days from onset to its resolution indicated by an absence of clinical signs, no fever and a normal white cell count.

### STATISTICAL EVALUATION

Each variable had two possibilities (Table 2) and the proportion of patients who recovered from cholangitis was computed for each of the two categories. Statistical significance of the difference between the two proportions obtained was tested by Chi-square after arcsein transformation of the proportions[6].

Statistical significance at the .05 level can only be achieved by having at least five patients in the smallest group. Thus all variables with less than five subjects in one of the two groups were not evaluated.

Patients who recovered from cholangitis were further evaluated to define a possible role for each of the variables in producing a shorter clinical course.

### RESULTS

Twenty five patients (86%) had a single episode of acute cholangitis; of these, twenty one (72%) recovered and 4 (14%) died of sepsis related to cholangitis lasting a median of one month. Four subjects suffered recurrences (14%).

Cholangitis occurred a median of 9 days from PTBD (range 1 day - 7 months), and lasted a median of 7 days (1–20 days). Sixteen patients died of progressive malignancy a median of 6 months from PTBD and 2 died of other causes after 7 and 8 months respectively; seven subjects are alive a median of 8 months after PTBD. Details concerning outcome and clinical course of cholangitis are reported in Table 3. Thirty-day mortality was 7% (2 patients, one of which died of neoplastic disease without sepsis).

Out of the large number of variables considered (Tables 1 and 2), only a few prognostic factors proved statistically related to a better prognosis in cholangitis: a stricture below the confluence of the two main ducts, a large catheter (10F or more in diameter) and a patient response suggested by an increase in body temperature to greater than 39 °C (Table 4).

All previously reported variables were also analyzed for correlation with the duration of cholangitis but none was found.

Bacteriological examination of the bile sample was performed in 13 patients at the onset of cholangitis: in one case Klebsiella was cultured alone, and in two cases only Pseudomonas species were grown; most frequently (70% of the cultured cases) the presence of enteric bacteria such as E. coli or Klebsiella species were associated with St. epidermis, Str. anhemoliticus, Str. alpha hemoliticus, Str. beta hemoliticus, Enterobacter species or Proteus mirabilis.

About half of the cholangitis sufferers, including those who may develop recurrences, show resolution within 5 months, while none will resolve if it lasts more than 10 months.

### DISCUSSION

Although some authors claim that the diagnostic and therapeutic role of PTBD is controversial[7], the technique has been extensively used after the early experience of Molnar and Stockum[8]. Several complications were reported, such as biliary

**Table 3** Clinical course and outcome of cholangitis after PTBD in neoplastic patients.

| *Outcome* | *N. pts* | *Median time from PTBD (range)* | *Occurence of cholangitis from PTBD (range)* | *Duration of cholangitis (range)* |
|---|---|---|---|---|
| awm | 7a | 8 mos<br>(1–15 mos) | 3 days<br>3 days–3 mos) | 6 days<br>(1–20 days) |
| dpm | 16b | 6 mos<br>(1–28 mos) | 20 days<br>(1 day–7 mos) | 7 days<br>(5–12 days) |
| doc | 2 | 8 mos<br>(7–8 mos) | —<br>(3–6 mos) | —<br>(3–7 days) |
| dbs | 4 | 1 mo<br>(1–4 mos) | 11 dys<br>(2–16days) | 1 mo<br>(15–80 days) |

awm=alive with malignancy
dpm=died of progressive malignancy
doc=died of other causes (heart attack and coagulopathy)
dbs=died of biliary sepsis
a: patients are alive with recurrent cholangitis after 8 and 16 months
b: patients survived 5 and 10 months with recurrent cholangitis

**Table 4** Statistically significant variables related to positive resolution of cholangitis.

| *Variables* | *N. patients* | *Resolved* | *p* |
|---|---|---|---|
| stricture above/at the confluence | 22 | 14 | 0.01 |
| stricture below/at the confluence | 7 | 7 | |
| first drainage <10F | 18 | 11 | 0.05 |
| first drainage >10F | 11 | 10 | |
| temperature at <39 | 22 | 14 | <0.01 |
| cholangitis >39 | 7 | 7 | |

leakage, bleeding, electrolyte and nutritional deficiencies, but sepsis related problems stand out. Cholangitis is a frequent complication, its prevalence ranging from 14.5% (2) to almost 50% (9–11) and it is more frequently observed in patients with malignancies (54%) than in those with benign disease (22%)[12].

It is well documented by Thompson[13] that neoplastic jaundice is more prone to cause recurrent cholangitis and more likely to be fatal than is the case with stones.

The availability of a large number of effective antibiotic drugs and biliary decompression makes the difference between the present mortality rate for cholangitis ranging from 9 to 40% (9,13–16) compared with the early work of Rogers[17] who reported a 100% mortality rate; despite this, biliary sepsis can cause septic shock and become a life-threatening condition. A careful technique followed by good care of the catheter, including flushing with saline to remove sludge and clots, helped in reducing the prevalence of septic episodes from 38.5% (4) in the 1982-84

INT series to 17.5% (1) in a 1986 update, and down to 15% described in this report. This figure compares well with the prevalence of cholangitis among patients with malignant biliary obstruction treated with endoscopic biliary endoprosthesis (17–20%) as recently reported[18,19].

Only a few factors have statistical importance in resolving cholangitis. The significant associations we have found can have only an indicative value due to the high number of comparisons performed: the anatomical site of the neoplastic stricture is one, since it is evident that low stenoses are better drained. Secondly, biological features such as an increase in body temperature (>39° C) are both statistically related to a more favourable outcome. Advantageous for a higher probability of resolving biliary sepsis is also a maximum flow through the catheter even in the presence of thick bile or debris when a larger catheter (10 F or more) is employed; this recommendation needs to be emphasized since it is the only variable we can effectively modify[20–22].

Repeated exchanges were also performed, both by the use of larger diameter catheters and repeated wash-outs with saline solution. It was also decided to administer antibiotics suggested by bacteriological tests instead of broad spectrum drugs. The intrabiliary route by direct injection through the catheter used for the administration of parenteral antibiotics. No significant advantage was achieved by these extensive nursing procedures in the present series. Several other factors aimed to improve bile flow might possibly be helpful in positively resolving cholangitis or in shortening its course (external opening, correct positioning of the side holes and brushings of the catheter)[23] but the limited number in the series does not allow statistical comparison.

We should consider PTBD as an effective procedure in most jaundiced neoplastic patients because of the increased cure rate of cholangitis and the low procedure-related mortality rate. This is also supported by a satisfactory overall median survival period of 6 months after PTBD.

### *References*

1. Cozzi, G., Ostinelli, C., Bellomi, M., Morosi, C., Pestalozza, C. and Severini, A. (1989) Il drenaggio biliare transepatico percutaneo: complicanze e loro trattamento. *La Radiologia Medica*, **77**, 399–404
2. Ferrucci, Jr., J.T., Mueller, P.R. and Harbin, W.P. (1980) Percutaneous transhepatic biliary drainage. Technique, results and applications. *Radiology*, **135**, 1–13
3. Berquist, T.H., May, G.R., Johnson, C.M., Adson, M.A. and Thistle, J.L. (1981) Percutaneous Biliary Decompression: Internal and external drainage in 50 patients. *AJR*, **136**, 901–906
4. Audisio, R.A., Bozzetti, F., Severini, A., Bellegotti, L., Bellomi, M., Cozzi, G., Pisani, P., Callegari, L., Doci, R. and Gennari, L. (1988) The occurrence of cholangitis after percutaneous biliary drainage: evaluation of some risk factors. *Surgery*, **103**, 507–512
5. Kauffmann, G.W., Roeren, Th., Friedl, P., Brambs, H.J. and Richter, G.M. (1990) Interventional radiological treatment of malignant biliary obstruction. *Europ. J. Surg. Oncol.*, **16**, 397–403
6. Miettinen, O.S. (1985) *Theoretical Epidemiology*, p. 143, New York: John Wiley & Sons
7. Leung, J.W.C., Cotton, P.B., Russel, R.C.G., Vallon, A.G. and Mason, R.R. (1983) Management of malignant jaundice at The Middlesex Hospital. *Br. J. Surg.*, **70**, 584–586
8. Molnar, W. and Stockum, A.E. (1974) Relief of obstructive jaundice through a percutaneous transhepatic catheter: a new therapeutic method. *Am. J. Roentgenol.*, **122**, 56–57
9. Dow, R.W., Lindenauer, S.M. (1969) Acute obstructive suppurative cholangitis. *Ann. Surg.*, **169**, 272–277
10. Saharia, P.C. and Cameron, J.L. (1976) Clinical management of acute cholangitis. *Surg. Gynecol. Obstet.*, **142**, 369–372

11. Chock, E., Wolfe, B.M. and Matolo, N.M. (1981) Acute suppurative cholangitis. *Surg. Clin. North Am.*, **61**, 885–892
12. Cohan, R.H., Illescas, F., Saeed, M., Pelmutt, L.M., Braun, S.D., Newman, G.E. and Dunnick, R. (1986) Infectious complications of percutaneous biliary drainage. *Invest. Rad.*, **21**, 705–709
13. Thompson, J.E., Tompkins, R.K. and Longmire, W.P. (1982) Factors in management of acute cholangitis. *Ann. Surg.*, **195**, 137–145
14. Saik, R.P., Greenburg, A.G. and Peskin, G.W. (1976) Cholecystostomy hazard in acute cholangitis. *J. Am. Med. Assoc.*, **235**, 2412–2413
15. Boey, J.H. and Way, L.W. (1980) Acute cholangitis. *Ann. Surg.*, **191**, 264–270
16. Saik, R.P., Greenburg, A.G., Farris, J.M. and Peskin, G.W. (1975) Spectrum of cholangitis. *Am. J. Surg.*, **130**, 143–150
17. Rogers, L. (1903) Biliary abscess of the liver with operation. *Brit. Med. J.*, **2**, 706–707
18. Shephard, H.A., Royle, G., Ross, A.P.R., Diba, A., Arthur, M. and Colin-Jones, D. (1988) Endoscopic biliary endoprosthesis in the palliation of malignant obstruction of the distal common bile duct: a randomized trial. *Brit. J. Surg.*, **75**, 1166–1168
19. Classen, M., Ossenberg, F.W. and Hagenmuller, F. (1985) Endoscopic-Radiologic examination and therapy in biliary tract disease. In: Wright, R., Millward-Sadler, G.H., Alberti, K.G.M.M., eds. *Liver and Biliary Disease*, p. 611. London: Baillier Tindall; W.B. Saunders Company
20. Severini, A., Cozzi, G., Bellomi, M., Frigerio, L. and Doci, R. (1987) A new set for transhepatic insertion of large caliber catheters for biliary drainage: technical and clinical report. *Tumori*, **73**, 635–638
21. Teplick, S.K., Haskin, P.H., Matsumoto, T., Wolferth, C.C., Pavlides, C.A. and Gain, T. (1984) Interventional Radiology of the Biliary System and Pancreas. *Surg. Clin. North Am.*, **64**, 87–119
22. Cotton, P.B. (1989) Nonsurgical palliation of jaundice in pancreatic cancer. *Surg. Clin. North Am.*, **69**, 613–626
23. Audisio, R.A. and Bozzetti, F. (1990) Cholangitis following percutaneous biliary drainage. *Ger. Med. Today*, **9**, 48–54

*(Accepted by S. Bengmark 20 May 1992)*

